# Computed Tomography–Based Body Composition in Patients With Ovarian Cancer: Association With Chemotoxicity and Prognosis

**DOI:** 10.3389/fonc.2021.718815

**Published:** 2021-11-16

**Authors:** Maria Del Grande, Stefania Rizzo, Gabriele Maria Nicolino, Ilaria Colombo, Lorenzo Rossi, Lucia Manganaro, Filippo Del Grande

**Affiliations:** ^1^ Service of Medical Oncology, Oncology Institute of Southern Switzerland, Ente Ospedaliero Cantonale (EOC), Bellinzona, Switzerland; ^2^ Istituto di Imaging della Svizzera Italiana (IIMSI), Ente Ospedaliero Cantonale, Lugano, Switzerland; ^3^ Facoltà di Scienze Biomediche, Università della Svizzera Italiana, Lugano, Switzerland; ^4^ Università degli Studi di Milano, Postgraduation School in Radiodiagnostics, Milan, Italy; ^5^ Department of Radiological, Oncological and Pathological Sciences, University of Rome Sapienza, Rome, Italy

**Keywords:** ovarian cancer, body composition, chemotherapy, visceral adipose tissue, skeletal muscle area

## Abstract

**Purpose:**

To assess the association between computed tomography (CT)-derived quantitative measures of body composition profiling and chemotherapy-related complications, in terms of dose reduction, premature discontinuation of chemotherapy, and cycle delays in patients with ovarian cancer. Secondary purposes were to evaluate associations between sarcopenia and survival, and to evaluate differences in body composition profiling at baseline and after neoadjuvant chemotherapy.

**Materials and Methods:**

The study population was retrospectively selected from a database of patients with newly diagnosed ovarian cancer (any stage) referred to our Institution between Feb 2011 and Mar 2020. Clinical data were recorded, and CT images at the level of the 3^rd^ lumbar vertebra were stored. By using specific software, skeletal muscle area (SMA), subcutaneous adipose tissue (SAT), visceral adipose tissue (VAT), and skeletal muscle density (SMD) were extracted. Skeletal muscle index (SMI) was then calculated. Statistical analysis was performed by logistic regression models to identify body composition features predictive of dose reduction, premature end of chemotherapy, and cycle delays. Kaplan-Meier analyses were performed to assess overall survival (OS) and progression-free survival (PFS). The log-rank test was used to determine differences in OS and PFS between sarcopenic and non-sarcopenic patients. Wilcoxon test was performed to compare body composition features before and after neoadjuvant chemotherapy (NACT).

**Results:**

Sixty-nine patients were included. A significant association was found between VAT and cycle delays (OR = 1.01, z = 2.01, 95% CI: 1.00–1.02, p < 0.05), between SMA and early discontinuation of chemotherapy (OR = 1.03, z = 2.10, 95% CI: 1.00–1.05, p < 0.05), and between mean SMD and cycle delays (OR = 0.92, z = −2.70, 95%CI: 0.87–0.98, p < 0.01). No significant difference emerged for OS in sarcopenic and non-sarcopenic patients, nor in CT body composition features before and after NACT.

**Conclusions:**

In ovarian cancer patients, CT-derived body composition profiling might predict the risk of chemotoxicity. In particular, VAT and SMD are associated with chemotherapy cycle delays, and SMA with early discontinuation of chemotherapy.

## Introduction

Epithelial ovarian cancer (OC) is the fifth cause of cancer death in the female population in developed countries, with 21,410 new cases estimated in the United States in 2021 (1). The current standard treatment for OC is primary cytoreductive surgery with complete resection of all macroscopic disease (2), followed by adjuvant platinum-based chemotherapy. When the disease distribution is considered not sufficiently amenable through primary cytoreduction, or the patient is not in proper conditions for upfront surgery, interval debulking surgery after neoadjuvant chemotherapy (NACT) is usually considered (3, 4).

The goal of chemotherapy dosing in both settings (adjuvant and neoadjuvant) is to find a balance between optimal efficacy and unacceptable toxicity, which could lead to drug dose delays, reductions, or a premature interruption of chemotherapy. In clinical practice, body surface area (BSA) has generally been used for chemotherapy dosing, because it is simple to calculate and it shows a general association with fat mass (5). However, BSA fails to detect differences in quantity and proportion of skeletal muscle and fat tissue. Sarcopenia refers to a skeletal muscle loss over two standard deviations below the mean of a young healthy adult group and can occur secondarily to a systemic disease, such as cancer (6). In many cancer types, sarcopenia has been associated with worse prognosis after curative surgery for localized disease, as well as during and after systemic therapy for advanced or metastatic disease (7–10). Indeed, sarcopenia and cachexia in cancer patients may be found in solid tumor, such as ovarian, especially in the metastatic setting, even though associated with high body mass index (11, 12).

If an association does exist between body composition profiling and chemotoxicity and/or prognosis, interventions might be introduced at diagnosis, such as physical activity exercise that can change skeletal muscle quantity and dysfunction, or even a combination of exercise, adequate nutrition (including proteins with high essential amino acids), and vitamin D supplementation, that has been demonstrated superior to exercise alone to increase muscle strength, muscle mass, and gait speed in sarcopenic patients (13).

Computed tomography (CT) represents a regular part of the standard management of staging procedures and follow-up in many cancer patients (14–16), including ovarian cancer, and it is considered an appropriate gold standard for non-invasive assessment of muscle quantity (17). Previous studies, designed with the specific aim to investigate the relationships between single cross-sectional abdominal image areas and the total volumes of skeletal muscle and adipose tissue across age and ethnicity groups in large samples, demonstrated that single abdominal skeletal muscle and adipose tissue areas are highly correlated with corresponding total body volumes in healthy individuals (18). In the specific setting of cancer patients, Mourtzakis et al. (19) provided evidence of a linear relationship between L3 and whole-body fat and fat-free compartments, demonstrating that CT imaging is a practical surrogate for whole-body imaging and it can be used with reasonable precision to predict whole-body measures for individual patients.

With this regard, advanced software allows the extraction of quantitative measurements from CT images, including skeletal muscle area (SMA), subcutaneous adipose tissue (SAT), skeletal muscle density (SMD), and visceral adipose tissue (VAT). Using these values, and knowing the height of the patients, it is possible to calculate the skeletal muscle index (SMI), which is a standard reference for sarcopenia (20).

To the best of our knowledge, no study has yet evaluated the association between SMI, SMA, SMD, VAT, and SAT, with the risk of chemotoxicity in OC patients treated with standard first-line platinum-based chemotherapy.

Therefore, the purpose of this study was to assess the association between CT-derived quantitative measures of body composition profiling and chemotherapy-related complications, in terms of dose reduction, premature discontinuation of chemotherapy, and cycle delays. Secondary purposes were to evaluate any association between sarcopenia and survival and to evaluate differences in body composition profiling at baseline and after neoadjuvant chemotherapy.

## Materials and Methods

### Patient Selection

The study population was retrospectively selected from a database of patients with newly diagnosed ovarian cancer referred to our institution between February 2011 and March 2020. The Ethics Committee approved this retrospective study with waiver of informed content (2020-01085). Inclusion criteria consisted of age ≥18 years, histologically confirmed high-grade serous ovarian cancer, first-line chemotherapy with or without debulking surgery, and availability in the picture archiving and communication system (PACS) of a CT scan or positron emitting tomography/computed tomography scan with iodinated contrast medium performed within 30 days before starting chemotherapy. Exclusion criteria consisted of previous or concurrent malignancy (the only exceptions were adequately treated carcinoma *in situ* of the cervix, non-melanomatous skin cancer, lobular or ductal carcinoma *in situ* of the breast, and any invasive cancer, other than breast cancer, in documented complete remission for ≥ 5 years); lost to follow-up in the first 6 months after starting the treatment; lack of information regarding chemotoxicity across the cycles of treatment; technical problems on the CT images, such as from metallic prostheses (21); documented refusal to the use of clinical data for research.

### Clinical Data Recording

The following clinical data were collected: age at diagnosis; weight and height to calculate the body mass index (BMI) and to further divide patients into three groups according to the World Health Organization (WHO) definition of obesity (22) into underweight (BMI <18.5), normal weight (BMI 18.5–24.9), overweight/obese (BMI >25); International Federation of Gynecology and Obstetrics (FIGO) stage; NACT, if any; dose reduction of any chemotherapy agent compared to first cycle; premature discontinuation of chemotherapy due to toxicity; cycle delays >2 weeks due to chemotherapy-induced adverse events; blood parameters within 30 days from the date of CT, including lactate dehydrogenase, albumin, hemoglobin, white blood cell count, lymphocyte count. Date of last contact, date of progression, and date of death were also recorded. Because of retrospective access to medical records, missing values were already present and not more recoverable. We decided not to exclude patients with some missing values to avoid selection bias.

### CT Data Extraction

**Figure 1:**
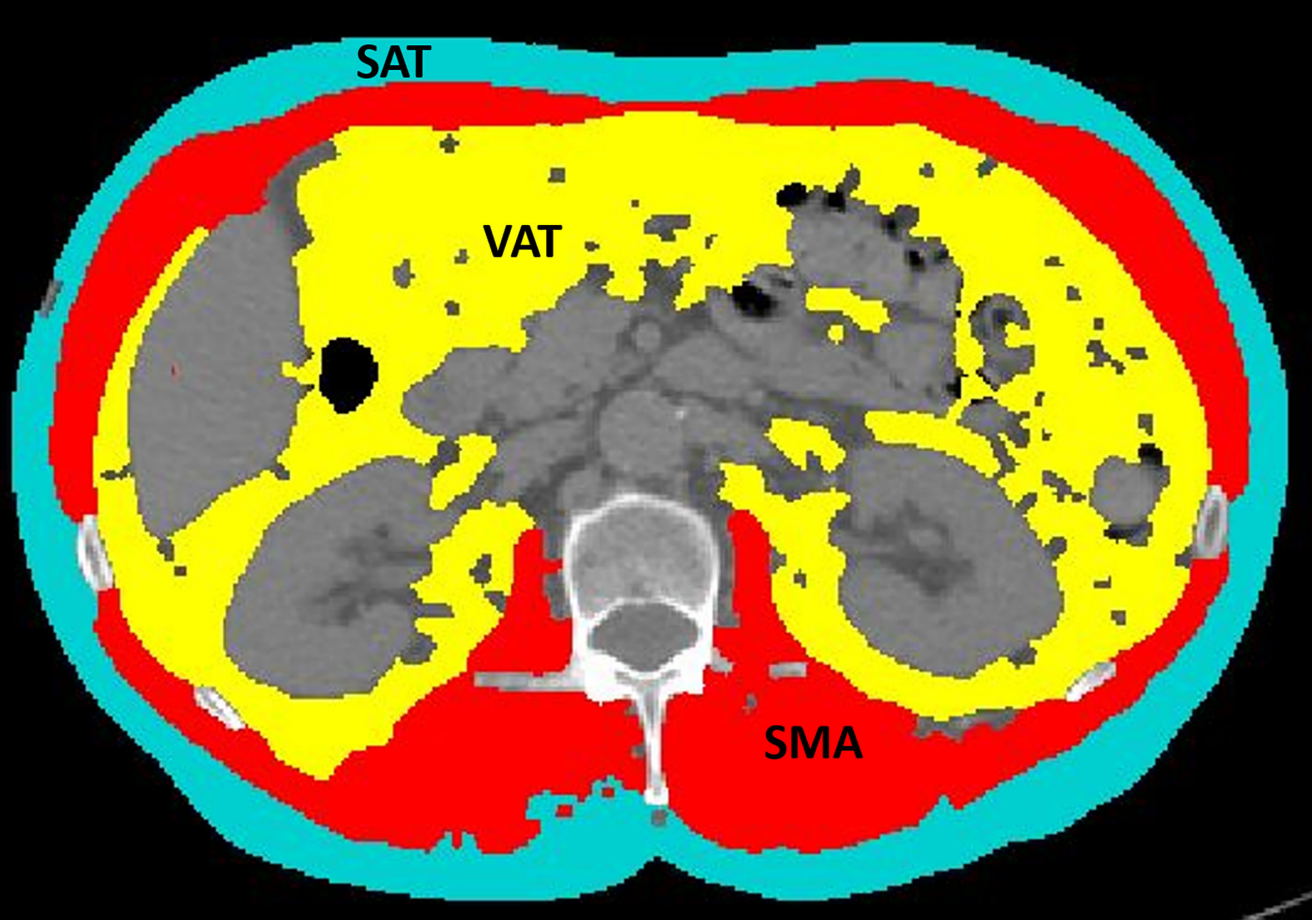
An example of segmentation of skeletal muscle area (red), visceral adipose tissue (yellow), and subcutaneous adipose tissue (light blue) from an axial image at the level of L3 of a CT scan performed as routine pretreatment staging. The colored parts are quantified as areas (cm^2^) by the software. The excluded parts include abdominal organs, such as liver, kidneys, and bowel (dark gray), as well as bones, such as vertebral body and ribs (light gray and white).

CT examinations were performed on different CT scanners at different institutions, but they were all available in digital format on our PACS. All the series used for extraction were acquired in the portal venous phase, after injection of contrast medium. A dedicated radiologist selected an axial CT image at the level of the third lumbar vertebra (L3), which was stored in digital imaging and communications in medicine format and then uploaded into the Slice-O-Matic software v 5.0 (Tomovision, Montreal, Canada). The morpho mode uses mathematical morphology to segment and edit the images. Mathematical morphology segmentation is done by computing the watershed of the gradient that gives a mosaic-like appearance to the image. Each region of this mosaic may then be filled with the appropriate tag value, corresponding to the tissue type (mainly muscle, subcutaneous fat, and visceral fat). The final step done by the software is to merge the areas of these regions together to give a quantification of the area in cm^2^. The region growing mode allows to threshold a specific region of the image. Once a threshold range is set up with upper and lower limits, all the pixels that fall within the range can be tagged. CT attenuation thresholds were −29 to 150 Hounsfield Units (HU) for skeletal muscle; −190 to −30 HU for subcutaneous adipose tissue; −150 to −50 HU for visceral adipose tissue. First, the morpho mode was applied; if it did not provide an easy way to make the segmentation, then the region growing mode was applied. By using either the morpho mode or the region growing mode, accurate segmentations of the muscle and fat were obtained, and the following quantitative measures were recorded: SMA (including the psoas, erector spinae, quadratus lumborum, transversus abdominis, external obliques, internal obliques, and rectus abdominis muscles) expressed in cm^2^; SMD expressed in HU; SAT expressed in cm^2^; and VAT expressed in cm^2^ (**Figure 1**). The lumbar SMI was calculated by normalizing SMA by square height (m^2^) and reported as cm^2^/m^2^. The sex-specific cutoff to define sarcopenia was SMI <41 cm^2^/m^2^ for women of any BMI (20). The overall survival (OS) was defined as the time between chemotherapy start and death from cancer or last visit. The progression-free survival (PFS) duration was defined as the time between the start of chemotherapy and disease progression.

### Statistical Analysis

Statistical analyses were performed using STATA16 (StataCorp^®^, College Station, TX, USA). Quantitative data were presented as mean ± standard deviation (SD) (minimum-maximum) or median and IQR if variables had non-normal distribution, verified through the Shapiro-Wilk test for normality. Qualitative data were reported as count and percentage of the total. Patients were classified as sarcopenic or non-sarcopenic using the abovementioned cutoff for SMI (20). Logistic regression models were performed to identify body composition features predictive of dose reduction or premature end of chemotherapy. Univariate models were fit to estimate odds ratios and 95% confidence intervals. Statistical significance was set at 5% (p < 0.05). Kaplan-Meier analyses were performed to assess OS and PFS. The log-rank test was used to determine differences between sarcopenic and non-sarcopenic patients in OS and PFS. Wilcoxon test was performed to compare body composition features before and after NACT given the non-normal distribution of the quantitative variables.

## Results

Sixty-nine patients were included with a mean age of 65.01 ± 11.45 years (43–88). Summary of patients’ characteristics, laboratory data, and CT quantitative measures of body composition are provided in **Table 1**. Most patients were staged as FIGO IIIC (n=38; 55%) and IV (n=11; 16%). Twenty-four (35%) patients received NACT before surgery. The laboratory tests before the first chemotherapy cycle showed that most patients had normal white blood cell count (7.27 ± 3.30 × 109/L), albumin (34.90 ± 7.70 g/L), hemoglobin (120.79 ± 18.15 g/L), and elevated lactate dehydrogenase (551.84 ± 355.14 U/L). Chemotherapy dose reduction occurred in 25 (36%) patients. Cycle delays (≥ 2 weeks) occurred in eight (12%) patients. Premature discontinuation of chemotherapy was needed in nine (13%) patients. Approximately 29% of patients were sarcopenic at diagnosis. Given the high standard deviation of VAT due to a high variation in body size of women included, we added a further subdivision of patients according to the WHO definition into underweight, normal weight, or overweight/obese (22).

**Table 1:** Baseline patient characteristics, laboratory data, and CT quantitative measures of body composition.

	All patients (n = 69)
**Patient characteristics: Demographics**	
**Age (year)**	65 ± 11.4 (43–88)
**BMI (kg/m^2^)**	24.9 ± 6.1 (16.9–52.8)
BMI range*	
***Underweight (<18.5)***	5 (7.69)
***Normal (18.5–22.9)***	23 (35.38)
***Overweight (23.0–24.9)***	8 (12.31)
***Obesity (≥25.0)***	29 (44.62)
**FIGO stage**	
*** IB***	1 (1%)
*** IIA***	1 (1%)
*** IIB***	2 (3%)
*** IIIA***	2 (3%)
*** IIIB***	5 (7%)
*** IIIC***	38 (55%)
*** IV***	20 (29%)
**Patient characteristics: Chemotherapy**	
**NACT no. of patients**	25 (36%)
**Dose reduction, no. of patients**	25 (36%)
**Early discontinuation of chemotherapy**	9 (13%)
**Cycle delays (≥ 2 weeks)**	8 (12%)
**Laboratory Tests**	
*** Lactate dehydrogenase (*10^2^ *U/L*)**	551.84 ± 355.14 (266–1,727)
***Albumin (g/L)***	34.90 ± 7.70 (14–46)
***Hemoglobin* (10^2^ *g/L*)**	120.79 ± 18.15 (74–163)
***White blood cell count (10^9^/L)***	7.27 ± 3.39 (1.6–19.3)
***Lymphocyte count (10^9^/L)***	1.41 ± 0.52 (0.26–2.87)
**Body Composition Parameters**	
***SAT* (×10^2^) (cm^2^)**	2.12 ± 1.27 (0.23-6.90)
***VAT* (×10^2^) (cm^2^)**	0.68 ± 0.60 (0–0.233)
***SMA* (×10^2^) (cm^2^)**	1.19 ± 0.26 (0.71–2.24)
***Mean SMD* (×10^2^) (HU)**	0.28 ± 0.16 (-0.18–0.56)
***SMI* (×10^2^) (*SMA/height^2^)***	0.47 ± 0.10 (0.26–0.81)
**Chemotherapy toxicity in sarcopenic patients**	
Sarcopenia**	20 (29%)
**Dose reduction**	8 (40%)
**Early discontinuation of chemotherapy**	3 (15%)
**Cycle delays**	2 (10%)

Quantitative data reported as mean ± standard deviation (minimum-maximum).

BMI, body mass index; FIGO, International Federation of Gynaecology and Obstetrics; NACT, neoadjuvant chemotherapy; SAT, subcutaneous adipose tissue; VAT, visceral adipose tissue; SMA, skeletal muscle area; SMD, skeletal muscle density; SMI, skeletal muscle index. *Missing values: BMI Range (n = 65).

**Optimal cutoff values determined by Martin (2013) (SMI < 41).

As reported in **Table 2**, considering the overall toxicity as the occurrence of at least one of the chemo-related complications, no significant association was found with SAT, VAT, mean SMD, SMA, and SMI. Nonetheless, analyzing each complication separately, we found a significant association between VAT and cycle delays (OR = 1.01, z = 2.01, 95% CI: 1.00–1.02, p < 0.05), between SMA and early discontinuation of chemotherapy (OR = 1.03, z = 2.10, 95% CI: 1.00–1.05, p < 0.05), and between mean SMD and cycle delays (OR = 0.92, z = −2.70, 95% CI: 0.87–0.98, p < 0.01). Looking specifically at sarcopenia (SMI<41), no significant differences emerged for dose reduction, early discontinuation of chemotherapy, and cycle delays between sarcopenic and non-sarcopenic patients.

**Table 2:** Univariate logistic regressions for the association of body composition values, toxicity, dose reduction, early discontinuation of chemotherapy, and cycle delays.

	Overall toxicity (n = 64)		Dose reduction (n = 64)		Early discontinuation of chemotherapy (n = 64)		Cycle delays (n = 60)	
	OR	z (p-value)	OR	z (p-value)	OR	z (p-value)	OR	z (p-value)
**SAT**	1.00	0.02 (0.984)	1.00	0.53 (0.597)	1.00	0.76 (0.446)	1.00	0.67 (0.506)
**VAT**	0.99	−0.19 (0.851)	1.00	0.55 (0.583)	1.00	0.80 (0.425)	1.01*	2.01 (0.044)
**SMA**	1.01	0.63 (0.526)	1.01	0.77 (0.441)	1.03*	2.10 (0.036)	1.02	1.57 (0.117)
**Mean SMD**	0.99	−0.85 (0.395)	0.98	−1.30 (0.192)	0.99	−0.62 (0.538)	0.92**	−2.70 (0.007)
**SMI**	1.00	0.19 (0.846)	1.01	0.30 (0.761)	1.03	1.00 (0.316)	1.02	0.66 (0.51)

Significance level lower than *0.05, **0.01; p-value reported in parentheses. Significant associations between VAT and SMD with cycle delays, and SMA and early discontinuation of chemotherapy. SAT, subcutaneous adipose tissue; VAT, visceral adipose tissue; SMD, skeletal muscle density; SMA, skeletal muscle area; SMI, skeletal muscle index.

**Figure 2:**
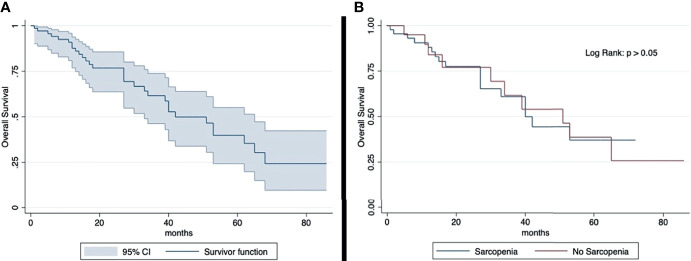
Kaplan-Meier curve showing overall survival for total sample **(A)** and by sarcopenia **(B)**. Median OS: 23 months (IQR: 29 months). 2-year OS rate: 77% (sarcopenic: 77%; non-sarcopenic: 78%); 5-year OS rate: 40% (sarcopenic: 39%; non-sarcopenic: 37%). The overall survival shows an overlap in the two groups divided according to sarcopenia.

**Figure 3:**
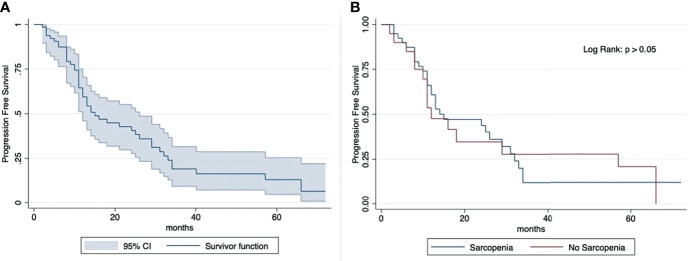
Kaplan-Meier curve showing progression-free survival for total sample **(A)** and by sarcopenia **(B)**. Median OS: 13 months (IQR: 17 months). 2-year PFS rate: 41% (sarcopenic: 35%; non-sarcopenic: 44%); 5-year PFS rate: 13% (sarcopenic: 21%; non-sarcopenic: 12%). The progression-free survival shows an overlap in the two groups divided according to sarcopenia.

The median OS was 23 months (IQR: 29 months). The 2-year and 5-year OS rates (2-year, 77%; 5-year, 40%) were similar in sarcopenic (2-year, 77%; 5-year, 39%) and non-sarcopenic (2-year, 78%; 5-year, 37%) patients (log-rank test: p > 0.05) (**Figure 2**). The median progression-free survival was 13 months (IQR: 17 months). The 2-year PFS rates and 5-year (2-year: 41%; 5-year: 13%) were similar in sarcopenic (2-year: 35%; 5-year: 21%) and non-sarcopenic (2-year: 44%; 5-year: 12%) patients (log-rank test: p > 0.05) (**Figure 3**).

No significant difference emerged in CT body composition features before and after neoadjuvant chemotherapy (**Table 3**).

**Table 3:** Body composition values before and after NACT (n = 25).

	Pre	Post	p-value*
**SAT (cm^2^) (×10^2^)**	1.44 (1.65)	1.64 (1.91)	0.165
**VAT (cm^2^) (×10^2^)**	0.64 (0.73)	0.43 (0.58)	0.442
**SMA (cm^2^) (×10^2^)**	1.21 (0.23)	11.45 (0.30)	0.072
**Mean SMD (HU) (×10^2^)**	0.21 (0.16)	0.28 (0.22)	0.149
**SMI (SMA/height^2^) (×10^2^)**	0.48 (0.12)	0.45 (0.92)	0.052

*Wilcoxon matched-pairs signed-rank test. Data were reported as median (IQR).

NACT, neoadjuvant chemotherapy; SAT, subcutaneous adipose tissue; VAT, visceral adipose tissue; SMA, skeletal muscle area; SMD, skeletal muscle density; SMI, skeletal muscle index; HU, Hounsfield Units.

## Discussion

In this study, we found significant associations between SMD and VAT with cycle delays, as well as between SMA and early discontinuation of chemotherapy, with body composition values measured as cross-sectional areas on a CT slice at the level of L3.

OC is the most lethal gynecological malignancy (1). The first-line treatment of OC is platinum-based chemotherapy following surgery as well as in the neoadjuvant setting. Chemotherapy dosing is commonly calculated using BSA, but conventional practice often involves capping a dose at a BSA of 2.0 or 2.2 m2 in an attempt to avoid overdosing (23). There is growing evidence suggesting that lean body mass correlates with drug clearance better than BSA, and this may play a role in predicting patient toxicities (24).

Body composition is associated with risk of toxicity-induced modifications of treatment in many types of tumors, such as breast (25), colon (26), and esophageal cancers (27). CT can provide estimates of lean muscle mass and adipose tissue as well as fat infiltration within the skeletal muscle (28). Magnetic resonance imaging (MRI) in body composition analysis shows a better soft tissue definition particularly of adipose tissue compared to CT, because fat shows short T1 and long T2 proton relaxation times, and this makes image segmentation of adipose tissues and skeletal muscle easier for the reader. Furthermore, an even improved segmentation of MRI fat and lean body mass may be produced using the “quantitative fat water imaging.” The basis for quantitative fat water imaging is fat water separated, or Dixon, imaging, where the different magnetic resonance frequencies of protons in fat and water are used for separating the two signals into a fat image and a water image (29, 30). However, the use of MRI is limited by the local availability and technical expertise, as well as by higher costs compared to CT. In this study, we performed body composition evaluation by CT-derived measures, because ovarian cancer patients undergo CT scans as preoperative evaluation and follow-up, being MRI is usually restricted to the characterization of undetermined adnexal masses at ultrasound (31).

Adipose tissue mostly consists of adipocytes, serves as an important reserve, and insulates the body from heat and cold. Many clinical factors influence the amount and distribution of the adipose tissue throughout the lifespan, as demonstrated by larger areas of VAT deposition in post-menopausal women. Additionally, muscle composition may be affected by the deposition of intermuscular fat (fat between skeletal muscle bundles and beneath the muscle fascia) and intramuscular fat (fat inside muscle fibers), which decrease the SMD at CT. OC patients, compared to patients with other malignancies, may suffer from changes in weight due to the presence of ascites, to the catabolism related to large tumor masses, and to the reduced food intake associated with bowel impairments (32). Therefore, a precise quantification of body composition profiling in these patients would need specific assessments for the different body compartments to find associations with toxicity profiles related to chemotherapy. Previous studies suggested that one cross-sectional image of CT scans at the level of the third lumbar vertebra may reliably represent individual’s body composition, including total body skeletal muscle, adipose tissues, and fat distribution (33, 34). Furthermore, with a specific attention to VAT, Shen et al. demonstrated that the strongest association with VAT volume across age and race groups in women was located 5 cm above L4-L5 (r=0.972), which is approximately the level of L3 (35).

VAT and SMD relate to fat distribution and deposits. Obese patients often show unpredictable responses to chemotherapy dosing (36), as obesity affects drug pharmacokinetics. The two most relevant pharmacokinetic parameters are the estimate of drug distribution into extravascular tissue and the drug clearance, and are both affected by obesity (5, 37–39). The association of VAT and SMD with the need for chemotherapy cycle delays is concordant with these findings. In agreement with our results, Ataseven et al. confirmed the importance of CT muscle density, showing that low muscle attenuation, but not SMI, was an independent risk factor for poor prognosis in OC patients, and this was more evident in patients with residual macroscopic tumor following debulking surgery (40). Accordingly, other studies showed the important role of SMD in OC patients, showing that low SMD is associated with poor prognosis (41–43). On the other hand, Prado et al. assessed the association of CT-based body composition values and dose-limiting toxicity, expressed as chemotherapy dose delays or interruptions, in patients with advanced relapsed OC undergoing treatment with pegylated liposomal doxorubicin (44). They suggested that certain patterns of body composition can predict and explain dose-limiting toxicities. Although their evaluation was dedicated to the overweight/obese group (n=48), patients who experienced dose-limiting toxicity had lower BMI and lower fat mass compared to those who did not experience dose-limiting toxicity.

In our cohort, we found that SMA was associated with early discontinuation of chemotherapy (OR = 1.03, z = 2.10, 95% CI: 1.00–1.05, p < 0.05), whereas sarcopenia, based on SMI, did not show significant associations with chemotherapy toxicities. Accordingly, in a cohort of 201 patients, 60% of which were sarcopenic, Staley et al. found no significant differences in chemotherapy management between the sarcopenic and non-sarcopenic groups, with 40.3% of the sarcopenic patients and 35.6% of non-sarcopenic patients requiring dose reduction (p = 0.58) (45).

Despite the association of sarcopenia with worse survival in other solid cancers, this association was not identified in our cohort of OC patients. Regarding association between body composition and OS, the literature on OC is not concordant. A recent meta-analysis showed a significant association between SMI and OS in OC (0.02; HR: 1.17, 95% CI: 1.03–1.33) (46), and results of a meta-analysis presented at a 2021 virtual congress showed that sarcopenia is highly associated with poor OS in OC patients (https://doi.org/10.1016/j.annonc.2021.05.577), whereas other authors show opposite results, with no association between SMI and OS (27, 30, 33). These conflicting results may partly be due to the lack of multivariate analyses, including other known risk factors for OS, such as complete primary debulking surgery and BRCA status.

In our cohort, 24/69 patients (35%) underwent NACT, and a specific evaluation of changes in body composition measurements at baseline CT and after NACT showed no significant changes. This result is discordant with other studies performed on gastric (47), colorectal (48), non-small-cell lung (49), and pancreatic cancers (50). This difference can be due to the specific setting of OC patients. Indeed, the majority of OC patients present with symptomatic ascites, and the treatment decision comes earlier compared to other advanced cancer types. Furthermore, the majority of OC patients show a quick response to the first-line treatment with carboplatin and paclitaxel, and therefore there is no further development of muscle consumption (51). Previous studies demonstrated that patients undergoing NACT were more likely sarcopenic compared to patients undergoing primary cytoreductive surgery (p = 0.04); however, no changes in body composition induced by NACT were found (52). Despite the lack of body composition changes in our subset of NACT patients, there is growing evidence that pre-habilitation protocols, as part of the enhanced recovery after surgery concept, may improve surgical outcomes, by bringing a fitter patient to surgery and decreasing treatment morbidity (53). Therefore, further studies, including lager cohorts selected in a prospective way (54), will provide deeper insights into the importance of changes in body composition before major surgery, also in the subset of NACT patients.

This study has several limitations. First, the lack of any significant association between sarcopenia (low SMI) and chemo-related complications might be due to the small sample size used to perform univariate logistic regressions. However, the literature on this topic is not univocal, and our cohort was quite homogeneous (first diagnosis and first-line chemotherapy), therefore consistent in this specific setting. Second, we did not perform a multivariate logistic regression, including nutritional diaries, appetite problems, muscle strength analysis, tumor stage, residual tumor at surgery, and chemotherapy approaches. Indeed, the pathophysiology of appetite problems in cancer patients is a complex process that involves many factors (55), as well as nutritional diaries do. However, since the design of this study was retrospective, the abovementioned data were not available. Prospective studies, also including pharmacokinetics data, might possibly help in understanding the reasons for associations between body composition and chemotoxicity. Finally, the results of semi-automatic segmentation may be slightly different from those obtained by automatic segmentation, and we did not perform the assessment with both techniques. However, since the SMD relies on density values, and the automatic segmentation works by predefined HU values, we decided not to exclude *a priori* any density values, and therefore we used just the semi-automatic segmentation, as it is more precise, albeit more prone to inter-reader variability.

In conclusion, this study demonstrates that in ovarian cancer patients, CT-derived body composition profiling may be associated with complications related to chemotherapy, VAT and SMD being associated with chemotherapy cycle delays, and SMA with early discontinuation of chemotherapy.

## Data Availability Statement

The raw data will be available under request to the corresponding author, after specific authorization by Swissethics. Requests to access the datasets should be directed to stefania.rizzo@eoc.ch.

## Ethics Statement

The studies involving human participants were reviewed and approved by Swissethics. Written informed consent for participation was not required for this study in accordance with the national legislation and the institutional requirements.

## Author Contributions

(I) Conception and design: All authors. (II) Administrative support: SR and FDG. (III) Provision of study materials or patients: MDG, GN, and SR. (IV) Collection and assembly of data: MDG, GN, and SR. (V) Data analysis and interpretation: MDG, SR, FDG. (VI) Manuscript writing: All authors. (VII) Final approval of manuscript: All authors. All contributed to the article and approved the submitted version.

## Conflict of Interest

The authors declare that the research was conducted in the absence of any commercial or financial relationships that could be construed as a potential conflict of interest.

## Publisher’s Note

All claims expressed in this article are solely those of the authors and do not necessarily represent those of their affiliated organizations, or those of the publisher, the editors and the reviewers. Any product that may be evaluated in this article, or claim that may be made by its manufacturer, is not guaranteed or endorsed by the publisher.
